# A Larger BAT Improves Metabolism but Whiffs on Safety

**DOI:** 10.1016/j.ebiom.2017.09.036

**Published:** 2017-09-28

**Authors:** Craig Blackstone

**Affiliations:** Neurogenetics Branch, National Institute of Neurological Disorders and Stroke, National Institutes of Health, Bethesda, MD, USA

Obesity is at epidemic levels in many countries worldwide, largely due to deleterious lifestyle and dietary factors. For instance, in the United States over a third of adults are obese and another third are overweight; childhood obesity is also disturbingly prevalent (Flegal et al., 2016). Given severe health consequences of obesity such as metabolic syndrome, type II diabetes, cancer, and cardiovascular disease, devising new means to reduce the incidence and severity of obesity is imperative. Much research focuses on modulating different types of adipose (fat) tissues, which each play key but distinct roles in regulating lipid metabolism, glucose homeostasis, thermogenesis, and inflammation (Peirce et al., 2014). White adipose tissue (WAT) stores excess energy in the form of triglycerides and secretes adipokines such as leptin and adiponectin; expansion of WAT (often dubbed “bad” fat) also shoulders much of the blame for obesity and its complications. The WAT-derived adipokines have been correlated with increased risks of neurodegenerative disorders such as dementia (Kiliaan et al., 2014), and other neurologic disorders are also associated with obesity (O'Brien et al., 2017).

Not all fat is considered bad, however. Brown adipose tissue (BAT) burns lipids to generate heat, and BAT has long been considered the “good” fat. In human newborns, abundant BAT is responsible for maintaining body temperature without the need for shivering. In adults, BAT is less abundant, but still found within a number of body regions. Though most prominent in supraclavicular and intrascapular regions, BAT is also found in paravertebral, mediastinal, para-aortic, suprarenal, and epineurial locations in adults. Individual BAT adipocytes differ from WAT adipocytes in having more iron-rich mitochondria and multiple lipid droplets in contrast to the single, large lipid droplet in WAT cells. BAT is also more vascularized than WAT, a feature that assists in dissipating heat and, when combined with the iron in its abundant mitochondria, confers its distinctive brown coloration (Peirce et al., 2014).

In addition to mediating the body's defense against cold via non-shivering thermogenesis, BAT secretes endocrine factors to modulate energy metabolism. For these reasons, BAT expansion presents a compelling means to counteract obesity. However, the possible deleterious effects of increasing BAT are not well understood. In *EBioMedicine*, Xiong et al. highlight one such example (Xiong et al., 2017). These authors investigated a mutant mouse (*Ad-Lkb1*) harboring an adipocyte-specific knock out of the tumor suppressor liver kinase B1 (LKB1), which is also a key regulator of lipid metabolism. In this context, the LKB1 kinase links cellular structure and energy utilization through activation of the AMP-activated protein kinase (AMPK) family of kinases which then inhibit the mTOR pathway, a central regulator of mammalian metabolism and physiology (Saxton and Sabatini, 2017). This adipocyte-specific knockout enlarged BAT in these mice, improving systemic metabolism (Shan et al., 2016). However, at 8 months of age these mice developed peripheral neuropathy and hind limb paralysis. Lkb1 was depleted in both WAT and BAT, but proinflammatory cytokines were only observed in BAT, with resulting inflammation in epineurial brown adipocytes around the sciatic nerve and subsequent damage. The neuropathy and inflammation were reduced by inhibiting mTOR genetically (via knock out) or pharmacologically (through treatment with rapamycin) (Xiong et al., 2017). Importantly, suppressing inflammation is not risk-free, since adipocyte inflammation is essential for healthy adipose tissue expansion and remodeling (Asterholm et al., 2014).

There are compelling links of inflammation to the neuropathy observed in these mice; inhibiting macrophages with clodrosome ameliorated the severity of the hind limb paralysis (Xiong et al., 2017). However, other mechanisms could also be in play, since both Lkb1 and mTOR have a plethora of cellular functions. Mice lacking Lkb1 in spinal cord, some brain regions, and endocrine pancreas develop hind-limb dysfunction and axon degeneration much earlier, at 7 weeks, with demyelination and macrophage infiltration of the spinal cord – but depletion of AMPK with the same strategy resulted in no obvious phenotype (Sun et al., 2011). Thus, it appears there may be a number of Lkb1-dependent pathways for related neurologic phenotypes. Furthermore, inhibiting LKB1 systemically may entail navigating a physiologic minefield, since this can drive cells into an energy/oxidative stress-induced state that can trigger activation of carcinogenic pathways to maintain cellular energy levels (Shorning and Clarke, 2016). Indeed, germ line mutations in *LKB1* are associated with Peutz–Jeghers polyposis and cancer syndrome, and somatic mutations are observed in sporadic forms of pulmonary, pancreatic and biliary cancers, as well as melanomas. LKB1 also regulates cell polarity through downstream kinases including MARKs and BRSKs, in addition to the nutrient utilization and cellular metabolism functions through the AMPK–mTOR pathway investigated by Xiong et al. (2017).

A key take-home point is that late-onset peripheral neuropathy is a potentially serious consequence of BAT-targeted obesity therapies. After all, obese patients (diabetics, certainly, but also those without frank diabetes) are already at significant risk for peripheral neuropathy (O'Brien et al., 2017), albeit through different mechanisms. In future studies, it will be important to determine whether other means for expanding BAT, such as converting WAT to BAT (Cao et al., 2011), cause similar adverse neurological effects.

## Disclosure

The author declares no conflicts of interest.
